# Microsurgical Clipping in Poor-Grade Aneurysmal Subarachnoid Hemorrhage (WFNS Grades 4–5) Patients from Hybrid Neurosurgeons’ Perspective: Clinical Profile and Functional Outcomes

**DOI:** 10.3390/brainsci16040364

**Published:** 2026-03-28

**Authors:** Miriam M. Moser, Luka Laub, Dorian Hirschmann, Anna Cho, Wei-Te Wang, Philippe Dodier, Gerhard Bavinzski, Karl Roessler, Arthur Hosmann

**Affiliations:** Department of Neurosurgery, Medical University of Vienna, 1090 Vienna, Austria

**Keywords:** aneurysmal subarachnoid hemorrhage, clipping, outcome, poor-grade, WFNS

## Abstract

**Highlights:**

**What are the main findings?**

- In poor-grade aSAH, microsurgical clipping is often required as a life-saving strategy due to severe hemorrhagic burden or unfavourable aneurysm anatomy.
- Primary decompressive craniectomy and delayed cerebral ischemia were associated with worse functional outcome, while meaningful recovery remains achievable despite high early morbidity.

**What are the implications of the main findings?**

- Even in critically ill poor-grade aSAH patients, microsurgical management can result in substantial functional recovery beyond hospital discharge.
- Early identification of severe hemorrhagic burden and prevention or management of delayed cerebral ischemia are crucial to optimize long-term functional outcomes.

**Abstract:**

**Background**: Aneurysmal subarachnoid hemorrhage (aSAH) remains a devastating neurological condition, with patients presenting with poor-grade aSAH having a particularly limited potential for recovery. Data on outcome trajectories after microsurgical clipping in this subgroup are scarce. The objective of this study was to analyze the functional outcomes in patients with poor-grade aSAH treated with microsurgical clipping, and to identify clinical factors associated with recovery. **Methods**: This retrospective study included 38 patients (median age 55 years; 60.5% female) with World Federation of Neurosurgical Societies (WFNS) grades 4–5, who underwent microsurgical clipping at a single tertiary care centre between 2016 and 2023. Functional outcome was assessed using the modified Rankin Scale (mRS) at hospital discharge and 6 months follow-up, and functional outcome was analyzed in relation to clinical variables (delayed cerebral ischemia (DCI), intracerebral hemorrhage (ICH), initial seizures, the need for decompressive craniectomy) using correlation and group comparison analyses. **Results**: The indication for microsurgical clipping was primarily driven by the need for ICH evacuation (50%) or by aneurysm configuration (47.5%). Microsurgical aneurysm clipping was performed on the day of hemorrhage in 25 patients (65.8%), with 16 patients (42.1%) undergoing immediate surgery following direct transfer from the emergency department to the operating theatre. ICH was present in 60.5% and IVH in 92.1%. Decompressive craniectomy was performed in 42.1%. DCI occurred in 21.6% of patients. In-hospital mortality was 15.8%, increasing to 22.6% at 6 months follow-up. Good functional outcome (mRS 0–2) was observed in 10.5% of patients at discharge and improved to 25.8% at 6 months. At hospital discharge, higher mRS scores were associated with the need for immediate aneurysm repair (*p* = 0.04), primary decompressive craniectomy (*p* = 0.02), and DCI (*p* = 0.006). Primary decompressive craniectomy (*p* = 0.04), reflecting greater disease severity, and DCI (*p* = 0.002) remained associated with worse functional outcome at 6 months. **Conclusions**: In poor-grade aSAH patients undergoing microsurgical clipping, mortality remains substantial; however, functional recovery may extend beyond hospital discharge. The need for immediate surgical intervention and primary decompressive craniectomy likely reflects a particularly severe hemorrhagic burden in patients and is associated with worse early functional outcomes, whereas DCI remains an important factor in overall functional recovery.

## 1. Introduction

Aneurysmal subarachnoid hemorrhage (aSAH) remains a devastating condition in general [1,2], but particularly in patients presenting with severe neurological impairment (World Federation of Neurosurgical Societies (WFNS) 4 or 5). Despite considerable progress in the domains of diagnosis, neurocritical care, and surgical techniques in recent decades, only approximately one-third of these patients achieve a favourable functional outcome [3]. It is noteworthy that advancements in functional outcome appear to have plateaued in recent years, a phenomenon that may be partly attributed to the increasing treatment of older patients and individuals presenting with more severe initial clinical conditions [3].

In contemporary neurovascular practice, the majority of patients with aSAH are treated using endovascular techniques wherever feasible [4,5]. However, a relevant subgroup of patients with poor-grade aSAH still requires microsurgical intervention because clinical or anatomical factors preclude endovascular therapy [6,7]. In patients with poor-grade aSAH, the decision to perform microsurgical clipping is often driven by the acute clinical scenario. A significant proportion of patients present with severe neurological deterioration or mass effect from intracerebral hemorrhage (ICH). In other cases, microsurgical clipping is indicated primarily due to aneurysm morphology. Even when patients present with a poor clinical condition, specific anatomical features of the aneurysm may preclude endovascular treatment, thereby necessitating microsurgical intervention [6,7]. Despite the fact that previous studies have evaluated treatment strategies and prognostic factors in poor-grade aSAH, the majority of contemporary series predominantly reflect endovascular management [8]. Consequently, outcome data specifically describing surgically selected cohorts, particularly those in whom microsurgical clipping represents the only viable treatment option, remain limited. Understanding outcomes in this particular subgroup is therefore important, as these patients continue to represent a complex population in routine neurovascular practice.

Secondary brain injury mechanisms, most notably delayed cerebral ischemia (DCI), play a crucial role in determining both short- and long-term functional outcome after aSAH [3]. In poor-grade patients undergoing microsurgical clipping, the relative contribution of disease severity, secondary injury, and clinical interventions within this surgically selected population remains insufficiently characterized.

The present study aims to evaluate functional outcomes in a cohort of poor-grade aSAH patients treated with microsurgical clipping at a hybrid neurovascular centre. The study places particular emphasis on clinical and radiological parameters reflecting hemorrhagic burden and secondary brain injury, including clinical presentation, ICH, primary and secondary decompressive craniectomy and DCI.

## 2. Methods

### 2.1. Population

This retrospective study includes 38 patients suffering from poor-grade aSAH (WFNS 4–5) whose ruptured aneurysms were treated by microsurgical clipping at the Medical University of Vienna between 2016 and 2023. The study was approved by the ethics committee of the Medical University of Vienna (EK-Nr. 1991/2023, date of approval: 21 December 2023)

Patients were characterized according to aSAH severity at the time of admission, using the WFNS grading scale together with clinical and radiological variables. These included seizures, loss of consciousness, extraventricular drainage, ICH, intraventricular hemorrhage (IVH), and the need for decompressive craniectomy. The parameters reflecting secondary brain injury included vasospasm, the necessity for spasmolysis, DCI, and cerebral infarction. Furthermore, the following data were collated: intensive care unit (ICU) length of stay, maximum transcranial Doppler (TCD) flow velocities during the patient’s hospitalization, TCD velocities on day 7, and the requirement for subsequent ventriculoperitoneal shunt placement.

The functional outcome was evaluated using the modified Rankin Scale (mRS) during routine clinical follow-up visits. These took place at the time of hospital discharge and at 6 months follow-up after the bleeding event. Assessments were conducted in person by treating clinicians as part of the standard clinical follow-up procedure. The clinicians were not blinded to the clinical variables. An mRS score of 0–2 was classified as a good functional outcome, and an mRS score of 3–6 was classified as a poor functional outcome.

### 2.2. Statistical Analysis

The statistical analysis was conducted using SPSS Statistics 30 (IBM Corp., Armonk, NY, USA) and MS Excel for Mac Version 16.105 (Microsoft, Redmond, WA, USA). Figures were generated using GraphPad Prism Version 10.6.1 (GraphPad Software, San Diego, CA, USA) for macOS. Continuous variables of patients’ characteristics are presented as median and interquartile range, while categorical variables are presented as absolute numbers and percentages. The functional outcome is presented in absolute numbers and percentages as well as graphically. The normal distribution of continuous variables was assessed using the Shapiro–Wilk test. The influence of primary clinical and radiological presentation (pupils, ICH, IVH, immediate surgery, microsurgical clipping and primary decompressive craniectomy in one surgery) on further complications (DCI, vasospasm, spasmolysis, TCD velocity, length of hospital stay, ventriculoperitoneal shunt placement) were analyzed using *t*-test or Mann–Whitney U test depending on the distribution of the variables and the Fisher exact test or χ^2^ test for categorical data, depending on the expected cell counts. Spearman correlation or Pearson correlation were used to calculate the correlations between ordinal and metric variables, depending on the distribution of variables. A two-sided *p*-value of <0.05 was considered statistically significant. Given the limited sample size and the number of variables explored, the statistical analyses were considered exploratory and hypothesis-generating rather than confirmatory. Consequently, the findings should be interpreted with caution.

## 3. Results

### 3.1. Population

**Figure 1:**
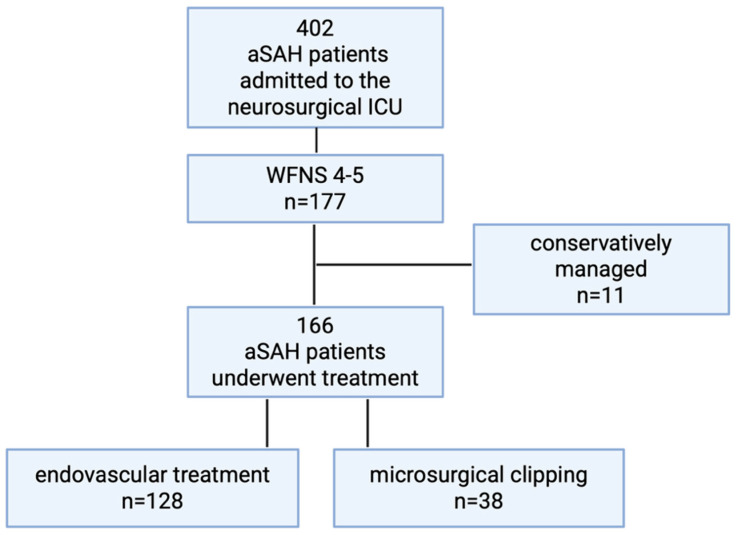
Patient flow diagram of aSAH population treated at our institution between 2016 and 2023; created in Biorender. Moser, M.M. (2026) https://BioRender.com.

During the study period between 2016 and 2023, a total of 402 patients were admitted to the neurosurgical intensive care unit (ICU) of our institution. Of these patients, 177 presented with poor-grade aSAH (WFNS grade 4–5). Of these, 38 patients underwent microsurgical clipping and were included in the present analysis, whereas 128 patients were treated using an endovascular approach and eleven patients were managed conservatively (Figure 1). Patients who were initially assessed in acute emergency settings and deemed not suitable for ICU-level neurosurgical care due to extremely poor prognosis were not captured in the dataset.

The clipping cohort consisted of 38 consecutive patients diagnosed with poor-grade aSAH who underwent microsurgical clipping at the Medical University of Vienna. The median age of the patients was 55 (IQR 49–60) years. Ruptured aneurysms in this cohort were predominantly located in the anterior circulation (94.7%), most frequently at the middle cerebral artery (50%), followed by the anterior communicating artery (31.6%). Detailed patient characteristics are summarized in Table 1.

Among the 128 patients treated endovascularly during the same period, the most frequent aneurysm location in this group was the anterior communicating artery (32%), followed by the posterior communicating artery (18.8%), the internal carotid artery (12.5%), and the basilar artery (10.9%). In contrast to the clipping cohort, a substantial proportion of aneurysms treated endovascularly were located in the posterior circulation (46.8%). Among the eleven conservatively managed patients in the neurosurgical ICU, the most frequent aneurysm location was the anterior communicating artery (36.4%), followed by the internal carotid artery and the posterior inferior cerebellar artery (18.2%, *n* = 2 each). All eleven patients died within three weeks of the hemorrhagic event.

### 3.2. Clinical Presentation and Surgical Management

The indication for microsurgical clipping was primarily driven by the need for ICH evacuation in 19/38 (50%) of cases and by aneurysm configuration in 18/38 (47.5%) of cases. In one patient, microsurgical clipping was performed because endovascular coiling was not feasible due to an occluded bifemoral bypass graft. Overall, 50% of the cohort underwent digital subtraction angiography (DSA) prior to microsurgical clipping, while the remaining 50% underwent computed tomography angiography only. Endovascular coiling was initially performed in two cases (10.5%). However, both patients subsequently developed rebleeding following the procedure and required microsurgical clipping.

The median time from the onset of hemorrhage to treatment of the aneurysm was 0 days (IQR 0–1). Twenty-five patients (65.8%) underwent microsurgical intervention on the day of hemorrhage, 10 patients (26.3%) within 1–3 days after aSAH and three patients received delayed treatment. Sixteen patients (42.1%) were transferred directly from the emergency department to the operating theatre, while the remaining patients were initially admitted to the neurosurgical ICU for clinical stabilization prior to surgery.

At the time of surgery, 78.9% of patients presented with impaired consciousness. In 63.2% of cases pupils demonstrated a reactive response, while 36.8% exhibited abnormal or non-reactive responses. Documented seizures prior to admission occurred in 39.5% of patients.

ICH was present in 60.5% of patients, with 50% requiring surgical evacuation of the hematoma. Patients with ICH were significantly more likely to be transferred directly to the operating room (60.9%) rather than initially stabilized in the neurosurgical ICU (39.1%; *p* = 0.005). Preoperative pupillary abnormalities were not found to be significantly associated with the presence of ICH (*p* = 0.33) (Supplementary Table S1).

IVH was present in 92.1% of the cohort.

In total, 16 patients (42.1%) underwent decompressive craniectomy. Of these, 13 patients underwent primary decompressive craniectomy during the microsurgical clipping procedure due to intraoperatively refractory intracranial hypertension and extensive brain swelling. Primary decompressive craniectomy was performed more frequently in patients presenting with abnormal pupillary status (60.5%) compared to those with normal pupillary status (38.5%; *p* = 0.035) (Supplementary Table S1). Three patients underwent secondary decompressive craniectomy due to delayed, medically refractory intracranial hypertension resulting from secondary brain swelling. Among the patients who underwent decompressive craniectomy, ICH was present in 93.8%.

In 97.4% of cases, an external ventricular drainage (EVD) was inserted, while one patient received parenchymal ICP monitoring only. During the subsequent follow-up, 39.5% of patients underwent ventriculoperitoneal shunting.

The median length of stay in the ICU was 30 days (IQR 21–39). Older age was significantly associated with prolonged ICU stay (*p* = 0.005) (Supplementary Table S1).

Baseline clinical and radiological factors, including age, sex, ICH or IVH, preoperative pupillary status, and aneurysm location, were not associated with secondary complications such as DCI, vasospasm, use of rescue spasmolysis, ventriculoperitoneal shunting, TCD velocities, or ICU length of stay (all *p* > 0.05). Decompressive (primary and secondary) craniectomy was significantly associated with ICH (*p* < 0.001), and TCD velocity (*p* = 0.03), but not with other secondary complications (Supplementary Table S1).

### 3.3. Functional Outcome of Poor-Grade aSAH Patients After Microsurgical Clipping

**Figure 2:**
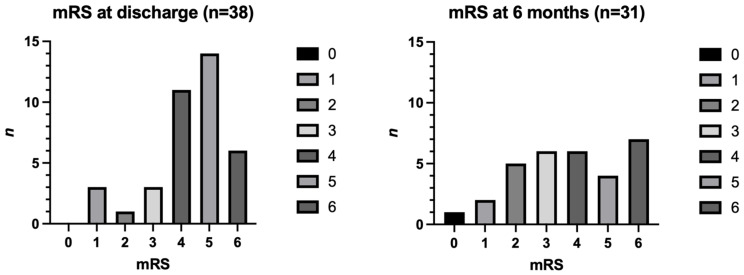
Distribution of functional outcome over time according to the modified Rankin Scale (mRS) at hospital discharge (*n* = 38) and at 6-month follow-up (*n* = 31). Seven patients were lost to follow-up between hospital discharge and the 6-month follow-up assessment. mRS = modified Rankin Scale.

The in-hospital mortality rate was 15.8% (six patients), with one additional death occurring during the 6-month follow-up period. This resulted in a cumulative mortality rate of seven patients over the study period (22.6%) (Figure 2).

At the time of discharge, 34 patients (89.4%) had a poor functional outcome, while four patients (10.5%) demonstrated a good recovery. At 6 months follow-up, seven patients (18.4%) were lost to follow-up. At this point, 23 patients (74.3%) had a poor functional outcome, while eight patients (25.8%) demonstrated a good functional outcome. The detailed functional outcomes are presented in Table 2 and Figure 2.

### 3.4. Determinants of Functional Outcome

Patients transferred directly from the emergency department to the operating theatre had a worse functional outcome at discharge (*p* = 0.04). Age, sex, pupillary status, and the occurrence of pre-admission seizures were not associated with mRS at any time point (all *p* > 0.05). ICH and IVH were not associated with worse functional outcome at discharge and 6 months follow-up (all *p* > 0.05). Primary decompressive craniectomy for refractory intracranial hypertension during the clipping procedure, reflecting severe initial brain injury, was associated with higher mRS at discharge (*p* = 0.02) and 6 months follow-up (*p* = 0.04). Maximum TCD velocities and the performance of rescue spasmolysis were not associated with mRS (all *p* > 0.05), nor the presence of radiological vasospasm (*p* > 0.05). Similarly, mRS did not differ significantly by the route of nimodipine administration (intravenous or oral). In contrast, the occurrence of DCI was associated with higher mRS at discharge (*p* = 0.006), and at 6 months follow-up (*p* = 0.002). Further details are summarized in Table 3 and Table 4.

In order to account for potential confounding, variables that demonstrated a significant association in univariable analyses (*p* < 0.05) were entered into a multivariable regression model. In light of the limited cohort size, the number of predictors was deliberately restricted to maintain model stability and reduce the risk of overfitting.

With regard to functional outcome at discharge, the multivariable model was statistically significant (f(3.33) = 7.20, *p* < 0.001) and explained 39.6% of the variance. DCI (β = 0.422, *p* = 0.004) and primary decompressive craniectomy (β = 0.361, *p* = 0.013) remained significant predictors of worse functional outcome, whereas direct transfer to surgery showed only a non-significant trend (*p* = 0.071). For mRS at the 6 months follow-up, the model remained significant (f(2.27) = 12.56, *p* < 0.001) and accounted for 48.2% of the variance. Both DCI (β = 0.621, *p* < 0.001) and primary decompressive craniectomy (β = 0.421, *p* = 0.006) remained independently associated with worse long-term functional outcome in this exploratory cohort.

The loss to follow-up rate at 6 months was 18.4% (7 of 38 patients), which may have influenced the interpretation of longitudinal outcome changes. The baseline characteristics of these patients were broadly comparable to the overall cohort, although they showed a tendency towards more severe hemorrhagic injury. The median age was 58 years (IQR 50–65) compared with 55 years (IQR 49–60) in the overall cohort. Intracerebral hemorrhage was present in 71.4% of patients lost to follow-up versus 60.5% in the full cohort, and intraventricular hemorrhage was present in all cases (100% versus 92.1%). Abnormal pupillary findings were observed in 42.9% of the lost-to-follow-up patients, compared with 36.8% in the overall study population. At hospital discharge, the majority of these patients had severe disability, which was comparable to the entire cohort (85.8% poor functional outcome vs. 89.4% poor functional outcome).

## 4. Discussion

This retrospective single-centre study of poor-grade aSAH patients treated with microsurgical clipping revealed that acute surgical intervention was frequently required and overall mortality remained substantial. A high hemorrhagic burden, particularly ICH, was strongly associated with the need for immediate surgical intervention and primary decompressive craniectomy, thereby identifying a subgroup of patients with particularly severe disease and worse early functional outcomes. DCI had a significant impact on the functional outcome at hospital discharge and remained a relevant determinant for highlighting the sustained impact of secondary brain injury over time within this surgically treated population.

### 4.1. Surgical Indications and Aneurysm Management

In the contemporary era of endovascular treatment, the majority of patients with poor-grade aSAH are treated using endovascular techniques, whenever anatomical circumstances permit [6]. Consequently, patients undergoing microsurgical clipping increasingly represent a surgically selected subgroup characterized by complex aneurysm morphology or the presence of space-occupying ICH requiring surgical decompression [7]. The present study therefore provides insight into the clinical characteristics, treatment indications, and outcomes of this specific subgroup and demonstrates that meaningful functional recovery remains achievable even among this surgically selected population of critically ill patients. These findings support the continued role of microsurgical clipping within modern multidisciplinary aneurysm care.

Moreover, these findings reflect real-world treatment decision-making in a hybrid neurovascular centre, where individualized therapy selection remains essential. In such cases, treatment decisions must take into account aneurysm morphology, hemorrhagic burden, and the patient’s clinical condition to determine the most appropriate intervention.

In the present cohort, the decision to perform microsurgical clipping was frequently driven by the acute clinical presentation rather than aneurysm morphology alone. A significant proportion of patients exhibited severe neurological deterioration, characterized by impaired consciousness, abnormal or non-reactive pupils, and rapidly progressive mass effect due to ICH. In such cases, the necessity for immediate surgical decompression and hematoma evacuation rendered microsurgical intervention the sole viable option [1,9].

Concurrently, aneurysm morphology also represented a significant factor in a considerable proportion of cases. Even among patients with poor neurological status, unfavourable anatomical characteristics of the aneurysm often precluded endovascular treatment [7]. This necessitated microsurgical clipping despite the critical clinical condition. In selected cases, the presence of additional technical or vascular access limitations further restricted the feasibility of endovascular options. Moreover, initial endovascular treatment did not invariably result in sustained occlusion of the aneurysm. In such cases, post-interventional rebleeding necessitated secondary microsurgical clipping to achieve definitive aneurysm exclusion.

It is important to note that treatment decisions were made by a dedicated team of hybrid neurosurgeons who had undergone training in both endovascular and microsurgical techniques [10,11,12,13,14]. This ensured that the selected strategy was guided by patient-specific clinical urgency and anatomical constraints rather than institutional preference.

Consequently, these acute clinical and radiological factors frequently necessitated immediate transfer from the emergency department to the operating room (42.1% of patients) for urgent aneurysm securing and, when required, acute decompression. In several cases, microsurgical clipping was combined with primary decompressive craniectomy to manage malignant brain swelling or refractory intracranial hypertension. These observations underscore that, in poor-grade aSAH, surgical strategy is primarily driven by the patient’s acute neurological status and the extent of hemorrhagic burden rather than solely by aneurysm anatomy.

### 4.2. Decompressive Craniectomy and Hemorrhagic Burden

Primary decompressive craniectomy was performed in patients with refractory intracranial hypertension or space-occupying ICH and was associated with worse functional outcome at 6 months follow-up. However, this association should be interpreted with caution, as it does not necessarily imply a causal detrimental effect of the procedure itself. Instead, decompressive craniectomy is more likely to represent a marker of severe initial brain injury [15]. In the present cohort, the strong association between ICH and decompressive craniectomy suggests that patients undergoing this procedure had a particularly high hemorrhagic burden. This hypothesis is further supported by the relatively high rate of decompressive craniectomy in the present cohort compared with previously published poor-grade populations (e.g., 11% in De Oliveira Manoel et al. [3] or 17.7% in Das et al. [16]).

These procedures are typically reserved for patients suffering from refractory intracranial hypertension, space-occupying ICH, or malignant cerebral edema, all of which are established predictors of poor functional outcome [17,18]. Importantly, patients who underwent primary decompressive craniectomy during the clipping procedure experienced worse functional outcomes across all follow-up time points, further underscoring that these interventions primarily reflect the severity of the initial disease. Consequently, decompressive craniectomy in this context should be interpreted primarily as a surrogate marker of disease severity rather than an independent causal determinant of outcome.

Brandecker et al. [19] reported a comparable rate of decompressive craniectomy in poor-grade aSAH, with 43.7% of patients undergoing primary decompressive craniectomy. In their cohort, ICH occurred in 44.8% overall and in 68% of patients requiring decompressive craniectomy. Notably, functional outcomes of patients with and without decompressive craniectomy were comparable, despite the poorer initial clinical status of those who underwent primary decompressive craniectomy [19]. This further supports the potential benefit of early decompressive craniectomy.

In the present cohort, the presence of ICH was significantly associated with the decision for decompressive craniectomy, likely reflecting mass effect and elevated intracranial pressure. It is well established that ICH can cause direct parenchymal damage and mass effect, leading to elevated intracranial pressure [20]. Previous studies have identified large hematomas or hemorrhages causing mass effect as predictors of poor functional outcome after aSAH [3,17]. Compared with previously published clipping-focused series, our cohort demonstrated a substantially higher prevalence of ICH. For instance, Das et al. [16] reported ICH in only 14% of cases in contrast to the 60% observed in the present cohort. Additionally, there was a slightly lower incidence of decompressive craniectomy (36.5% vs. 42.1%). These differences likely reflect the substantial hemorrhagic burden observed within our surgically treated cohort. In our cohort, the evacuation of hematoma was a frequent primary indication for microsurgical intervention.

In the present study, ICH was not associated with worse functional outcome at discharge or at the 6 months follow-up, suggesting that, in patients with already severe global brain injury, the incremental effect of ICH may be outweighed by the overall disease burden. In this context, the severity of the initial hemorrhagic insult and subsequent secondary complications appear to be the predominant determinants of long-term functional outcome, rather than the presence of ICH alone.

### 4.3. Baseline Characteristics: Age and Pre-Admission Seizures

In addition to the presence of hemorrhagic burden and surgical interventions, baseline patient characteristics and early neurological features may influence functional outcome assessment.

The mean age in the cohort was comparable to that of other poor-grade aSAH populations [3,21]. Within this severely affected group, age appeared to have a limited influence on functional recovery, as functional outcomes were primarily determined by the severity of the initial hemorrhage and subsequent complications. Nevertheless, the data demonstrate that older patients exhibited significantly longer stays in the ICU, consistent with previous reports [22].

In addition, pre-admission seizures were not associated with functional outcome at any time point, indicating that seizures do not constitute an independent determinant of secondary brain injury or long-term recovery in poor-grade aSAH. Seizures may have a transient effect on the initial neurological examination, potentially leading to an overestimation of clinical severity. However, the absence of improved functional outcomes in patients with seizures argues against a significant prognostic bias and suggests that these events likely reflect early cortical irritation rather than a reversible cause of neurological impairment, consistent with prior reports in severe hemorrhagic strokes [23].

### 4.4. Delayed Cerebral Ischemia and Secondary Complications

Following the acute phase, secondary complications such as DCI can significantly influence the trajectory of neurological recovery, particularly during the early post-hemorrhagic period. The association between DCI and significantly worse functional outcome at hospital discharge underscores its role as a critical early complication and confirms the pronounced impact of secondary ischemic injury on early neurological recovery, consistent with previous research [24]. The sustained association at later follow-up time points indicates that the negative effect of DCI on functional outcome persists beyond the early recovery phase [24].

### 4.5. Prognosis

Overall, in-hospital and 6-month mortality rates in our cohort were 15.8% and 22.6%, respectively. These rates are substantially lower than the up-to-60% mortality rates reported in other poor-grade aSAH cohorts [25]. This lower mortality is likely a reflection of the aggressive, intervention-oriented management strategy, which included prompt microsurgical clipping, hematoma evacuation, and primary decompressive craniectomy, as well as careful patient selection. It is important to note that the present study includes only patients who underwent microsurgical clipping and does not capture those managed conservatively, a group that typically exhibits substantially higher mortality rates [25]. Consequently, the outcomes presented herein predominantly reflect those achievable in a surgically treated and carefully selected subgroup of patients with poor-grade aSAH. Therefore, the observed mortality cannot be attributed solely to the surgical strategy itself. Instead, it is more probable that this outcome is indicative of a combination of factors, including treatment selection, centre-specific expertise, and the overall composition of this relatively small cohort.

In a previous study of 257 patients with poor-grade aSAH, poor functional outcome at hospital discharge (GOS 1–3) was observed in 76.9% of treated patients, compared to 94.4% in untreated patients, with persistent differences at 3 months (48% vs. 93.9%) [25]. The present findings are consistent with this trend, demonstrating gradual improvement over time despite the profound initial neurological impairment.

In a cohort of patients with poor-grade aSAH who were treated with either microsurgical clipping or endovascular coiling, Lu et al. highlighted the importance of early aneurysm repair, showing that treatment delays of 3–10 days after hemorrhage were associated with an increased risk of delayed cerebral ischemia and poor functional outcome. Subgroup analyses further demonstrated that advanced age and the presence of intraventricular hemorrhage on initial imaging were associated with a higher risk of worse outcome [26]. The findings of the present cohort are broadly consistent with these observations, as a significant proportion of patients (92.1%) received treatment within three days after hemorrhage (Table 1).

These observations highlight the possibility of delayed neurological recovery beyond the early post-acute phase, even in patients with severe impairment. However, due to the retrospective design of the study, it must be acknowledged that a degree of follow-up bias may have influenced the results. Patients with either very poor or very favourable functional outcomes may have been lost to follow-up, potentially influencing the observed recovery trajectory.

## 5. Limitations

Several limitations of this study should be acknowledged. Firstly, the retrospective design is inherently associated with incomplete data capture and potential selection bias, particularly with regard to long-term follow-up, which was unavailable for a substantial proportion of patients. Importantly, patients with the most severe neurological impairment may have been less likely to attend follow-up assessments or to have functional outcomes documented, potentially leading to an underrepresentation of unfavourable long-term functional outcomes. Despite the fact that functional status at discharge did not differ substantially in the subgroup of patients who were lost to follow-up, it is possible that these patients may nevertheless have constituted a subgroup with slightly greater initial hemorrhagic burden.

Secondly, the relatively small sample size (*n* = 38) limits statistical power and increases the risk of type I error when multiple variables are explored. Therefore, the reported associations should be interpreted as exploratory and hypothesis-generating rather than definitive evidence of causal relationships Thirdly, the single-centre design may limit the generalizability of the findings, as treatment strategies, patient selection, and perioperative management may differ across institutions.

Finally, the cohort consisted exclusively of poor-grade aSAH patients treated with microsurgical clipping, representing a highly selected population in whom endovascular treatment was not feasible. While this limits direct comparability with mixed or endovascular cohorts, it also highlights the relevance of the findings for a clinically important but underrepresented subgroup.

## 6. Conclusions

In poor-grade aSAH, microsurgical clipping is frequently required in the acute setting due to severe neurological deterioration or mass effect from ICH. The necessity for primary decompressive craniectomy reflects a substantial hemorrhagic burden and a severe initial disease presentation. Secondary complications, most notably DCI, were shown to significantly influence functional outcome over time. Within this surgically selected population, a subset of patients undergoing microsurgical clipping demonstrated gradual functional recovery during follow-up despite the overall high morbidity associated with poor-grade aSAH. These findings underscore the potential for substantial recovery in carefully selected patients treated surgically in a contemporary hybrid neurovascular setting.

## Figures and Tables

**Table 1:** Patients‘ characteristics.

Characteristics	
Age (years; median)	55 (IQR 49–60)
Height (m; median)	1.7 (IQR 1.65–1.80)
Weight (kg; median)	72.5 (IQR 65–85)
BMI (median)	25 (IQR 22.9–27.7)
Sex	
Female	23 (60.5%)
Male	15 (39.5%)
WFNS	
4	15 (39.5%)
5	23 (60.5%)
Initial loss of consciousness	30 (78.9%)
Seizures prior to admission	15 (39.5%)
Localisation of ruptured aneurysm	
Internal carotid artery (ICA) C6	1 (2.6%)
Middle cerebral artery (MCA)	19 (50%)
Anterior communicating artery (AcomA)	12 (31.6%)
Pericallosal artery	3 (7.9%)
Posterior communicating artery (PcomA)	2 (5.3%)
Not identifiable (AcomA or PcomA)	1 (2.6%)
Time from aneurysm rupture to treatment:	
Acute surgery (0 days)	25 (65.8%)
Early surgery (1–3 days)	10 (26.3%)
Delayed surgery (4–21 days)	2 (5.3%)
Late surgery (>21 days)	1 (2.6%)
Time from aneurysm rupture to treatment (days; median)	0 (0–1)
Direct transfer to OR from emergency room	16 (42.1%)
Transfer to and stabilizing patient at the ICU	22 (57.9%)
Primary decompressive craniectomy during clipping procedure	13 (34.2%)
Decompressive craniectomy	16 (42.1%)
Pupils at time of surgery	
abnormal (anisocoria/wide/non-reactive, etc.)	14 (36.8%)
normal	24 (63.2%)
Intracerebral hemorrhage (ICH)	23 (60.5%)
ICH requiring hematoma evacuation	19 (50%)
Intraventricular hemorrhage (IVH)	35 (92.1%)
External ventricular drain (EVD)	37 (97.4%)
Delayed cerebral ischemia (DCI)	8 (21.6%)
Postoperative infarction other than DCI	22 (57.9%)
Vasospasm	21 (55.3%)
Maximum transcranial Doppler (TCD) velocity (cm/s, median)	148 (IQR 115–196)
TCD velocity at day 7 after aSAH (cm/s, median)	90 (IQR 61–129)
Spasmolysis	9 (24.3%)
ICU length of stay (days; median)	30 (21–39)
Ventriculoperitoneal shunt	15 (39.5%)

**Table 2:** Functional outcome after poor-grade aSAH and microsurgical aneurysm clipping. mRS = modified Rankin Scale.

Functional Outcome	mRS at Discharge (*n* (%))	mRS at 6 Months Follow-Up (*n* (%))
0	0 (0)	1 (3.2)
1	3 (7.9)	2 (6.5)
2	1 (2.6)	5 (16.1)
3	3 (7.9)	6 (19.4)
4	11 (28.9)	6 (19.4)
5	14 (36.8)	4 (12.9)
6	6 (15.8)	7 (22.6)

**Table 3:** Functional outcome at discharge was assessed using the modified Rankin Scale (mRS). Group comparisons were performed using the Mann–Whitney U test, and correlations using Spearman’s rank correlation coefficient (ρ). *p*-Values reaching statistical significance are presented in bold.

Variable	mRS at Discharge
Age	Spearman ρ = −0.11, *p* = 0.50
Direct transfer to OR	Mann–Whitney U = 109.5, ***p* = 0.041**
Abnormal pupils before surgery (wide, non-reactive)	Mann–Whitney U = 132.5, *p* = 0.263
Seizure before admission	Mann–Whitney U = 123, *p* = 0.124
Intracerebral hemorrhage (ICH)	Mann–Whitney U = 131, *p* = 0.20
Intraventricular hemorrhage (IVH)	Mann–Whitney U = 22, *p* = 0.09
Decompressive craniectomy (primary combined surgery with clipping)	Mann–Whitney U = 89.5, ***p* = 0.019**
Decompressive craniectomy (any time during ICU stay)	Mann–Whitney U = 122.5, *p* = 0.099
Radiological vasospasm	Mann–Whitney U = 162.5, *p* = 0.63
Delayed cerebral ischemia (DCI)	Mann–Whitney U = 44.5, ***p* = 0.006**
Spasmolysis	Mann–Whitney U = 106.5, *p* = 0.39
Route of Nimodipine administration (intravenous vs. oral)	Mann–Whitney U = 123.5, *p* = 0.57
Maximum TCD velocity	Spearman ρ = 0.08, *p* = 0.62
TCD velocity day 7	Spearman ρ = 0.10, *p* = 0.59
ICU length of stay	Spearman ρ = 0.04, ***p* = 0.82**

**Table 4:** Functional outcome at 6 months follow-up was assessed using the modified Rankin Scale (mRS). Group comparisons were performed using the Mann–Whitney U test, and correlations using Spearman’s rank correlation coefficient (ρ). *p*-Values reaching statistical significance are presented in bold.

Variable	mRS at 6 Months Follow-Up
Age	Spearman ρ = 0.18, *p* = 0.35
Direct transfer to OR	Mann–Whitney U = 75.5, *p* = 0.091
Abnormal pupils before surgery (wide, non-reactive)	Mann–Whitney U = 81.5, *p* = 0.23
Seizure before admission	Mann–Whitney U = 100, *p* = 0.564
Intracerebral hemorrhage (ICH)	Mann–Whitney U = 96, *p* = 0.39
Intraventricular hemorrhage (IVH)	Mann–Whitney U = 14, *p* = 0.06
Decompressive craniectomy (primary combined surgery with clipping)	Mann–Whitney U = 49, ***p* = 0.037**
Decompressive craniectomy (any time during ICU stay)	Mann–Whitney U = 74.5, *p* = 0.08
Radiological vasospasm	Mann–Whitney U = 75, *p* = 0.09
Delayed cerebral ischemia (DCI)	Mann–Whitney U = 19, ***p* = 0.002**
Spasmolysis	Mann–Whitney U = 67, *p* = 0.16
Route of Nimodipine administration (intravenous vs. oral)	Mann–Whitney U = 42.5, *p* = 0.22
Maximum TCD velocity	Spearman ρ = 0.17, *p* = 0.38
TCD velocity day 7	Spearman ρ = 0.24, *p* = 0.26
ICU length of stay	Spearman ρ = 0.24, ***p* = 0.20**

## Data Availability

The data presented in this study are available on request from the corresponding author due to containing information that could compromise the privacy of research participants.

## Supplementary Materials

The following supporting information can be downloaded at: https://www.mdpi.com/article/10.3390/brainsci16040364/s1, Table S1: Impact of initial clinical and radiological factors. DCI = delayed cerebral ischemia, VPS = ventriculoperitoneal shunt, TCD = transcranial Doppler, ICU = intensive care unit.
