# Supplementary material for: Microsurgical Clipping in Poor-Grade Aneurysmal Subarachnoid Hemorrhage (WFNS Grades 4–5) Patients from Hybrid Neurosurgeons’ Perspective: Clinical Profile and Functional Outcomes
Source: Brain Sci. 2026 Mar 28;16(4):364. doi: 10.3390/brainsci16040364 (PMC13115265; doi:10.3390/brainsci16040364)
Supplement: Supplementary file 1 [file brainsci-16-00364-s001.zip › brainsci-4188833-supplementary.pdf]

Supplementary Table S1. Impact of initial clinical and radiological factors. DCI = delayed cerebral ischemia, VPS = ventriculoperitoneal shunt, TCD = transcranial doppler, ICU = intensive care unit.

Parameter	Outcome	Result
**Age**	DCI	t(35) = 0.38, p = 0.71 (55 ± 12 years no DCI vs. 53 ± 9 years DCI)
	Radiological vasospasm	t(36) = 1.1, p = 0.30 (56 ± 13 years no vasospasm vs. 53 ± 9 years with vasospasm)
	Spasmolysis	t(36) = 0.8, p = 0.45 (55 ± 11 years no spasmolysis vs. 52 ± 10 years spasmolysis)
	VPS implantation	t(36) = -1.3, p = 0.20 (52 ± 9 years with no VPS vs. 58 ± 13 years with VPS implantation)
	Max. TCD velocity	Pearson r = -0.28, p = 0.09
	ICU length of stay	**Spearman ϱ = 0.44, p = 0.005**
**Sex**	DCI	Fisher exact test, p = 1.00
	Radiological vasospasm	Fisher exact test, p = 0.51
	Spasmolysis	Fisher exact test, p = 0.27
	VPS implantation	Fisher exact test, p = 0.74
	ICU length of stay	Mann–Whitney U = 165.6 p = 0.83
	Max. TCD velocity	t(36) = 1.9, p = 0.07 (128 ± 45 cm/s in men vs. 160 ± 56 cm/s in women)
**Seizures before admission**	ICH	Fisher exact test, p = 0.51
**Intracerebral hemorrhage (ICH)**	DCI	Fisher exact test, p = 0.69
	Radiological vasospasm	Fisher exact test, p = 0.55
	Spasmolysis	Fisher exact test, p = 0.27
	VPS implantation	Fisher exact test, p = 0.51
	Max. TCD velocity	t(36) = -0.9, p = 0.40 (154 ± 55 cm/s with ICH vs. 138 ± 53 cm/s without ICH)
	ICU length of stay	Mann Whitney U = 143, p = 0.38
**Abnormal pupils (anisocoria / wide, non-reactive)**	Primary decompressive craniectomy	Fisher exact test, **p = 0.035**
	ICH	Fisher exact test, p = 0.33
	DCI	Fisher exact test, p = 1.00
	Radiological vasospasm	Fisher exact test, p = 0.32
	Spasmolysis	Fisher exact test, p = 1.00
	VPS implantation	Fisher exact test, p = 0.10
	ICU length of stay	Mann–Whitney U = 143, p = 0.45
	Max. TCD velocity	t(36) = 0.11, p = 0.91 (147 ± 56 in patients with wide non-reactive pupils at time of surgery vs. 149 ± 54 cm/s in patients with normal pupils at time of surgery)
**Decompressive craniectomy**	ICH	**$\chi^2(1)$ = 11.96, p < 0.001, Fisher exact test p < 0.001**
	DCI	Fisher exact test, p = 1.00
	Radiological vasospasm	Fisher exact test, p = 0.20
	Spasmolysis	Fisher exact test, p = 0.45

	VPS implantation	Fisher exact test, p = 0.18
	Max. TCD velocity	t(36) = -2.2, **p = 0.03** (170 ± 50 cm/s in patients with decompressive craniectomy vs. 132 ± 52 cm/s in patients without decompressive craniectomy)
	ICU length of stay	Mann–Whitney U = 173.5, p = 0.94
**Intraventricular hemorrhage (IVH)**	Primary decompressive craniectomy	Fisher exact test, **p = 0.005**
	DCI	Fisher exact test, p = 1.00
	Radiological vasospasm	Fisher exact test, p = 0.58
	Spasmolysis	Fisher exact test, p = 1.00
	VPS implantation	Fisher exact test, p = 1.00
	Max. TCD velocity	t(36) = -1.5, p = 0.15 (104 ± 39 cm/s when no IVH was presen vs. 152 ± 54 cm/s when IVH was present)
	ICU length of stay	Mann–Whitney U = 49, p = 0.85
**Aneurysm location**	DCI	Fisher exact test, p = 0.61
	Radiological vasospasm	Fisher exact test, p = 0.11
	Spasmolysis	Fisher exact test, p = 0.68
	VPS implantation	Fisher exact test, p = 0.84
	Max. TCD velocity	Kruskal–Wallis-Test, H(5) = 8.1, p = 0.15
	ICU length of stay	Kruskal–Wallis-Test, H(5) = 3.4, p = 0.64
